# Meal Enjoyment and Tolerance in Women and Men

**DOI:** 10.3390/nu11010119

**Published:** 2019-01-08

**Authors:** Hugo Monrroy, Teodora Pribic, Carmen Galan, Adoracion Nieto, Nuria Amigo, Anna Accarino, Xavier Correig, Fernando Azpiroz

**Affiliations:** 1 Digestive System Research Unit, University Hospital Vall d’Hebron, Centro de Investigación Biomédica en Red de Enfermedades Hepáticas y Digestivas (Ciberehd), Departament de Medicina, Universitat Autònoma de Barcelona, 08193 Bellaterra (Cerdanyola del Vallès), Spain; hmonrroy@gmail.com (H.M.); teodora.pribic@gmail.com (T.P.); cgalanhidalgo@gmail.com (C.G.); anietoruiz7@gmail.com (A.N.); aaccarino@telefonica.net (A.A.); 2 Centro de Investigación Biomédica en Red de Diabetes y Enfermedades Metabólicas (Ciberdem), Metabolomics Platform, IISPV, Universitat Rovira i Virgili, 43002 Tarragona, Spain; namigo@biosferteslab.com (N.A.); xavier.correig@urv.cat (X.C.); 3 Biosfer Teslab S.L., 43201 Reus, Spain

**Keywords:** meal ingestion, post-prandial sensations, hedonic response, homeostatic sensations, gender differences, metabolomic response

## Abstract

Various conditioning factors influence the sensory response to a meal (inducible factors). We hypothesized that inherent characteristics of the eater (constitutive factors) also play a role. The aim of this proof-of-concept study was to determine the role of gender, as an individual constitutive factor, on the meal-related experience. Randomized parallel trial in 10 women and 10 men, comparing the sensations before, during, and after stepwise ingestion of a comfort meal up to full satiation. Comparisons were performed by repeated Analysis of Covariance (ANCOVA) measures. During stepwise ingestion, satisfaction initially increased up to a peak, and later decreased down to a nadir at the point of full satiation. Interestingly, the amount of food consumed at the well-being peak was lower, and induced significantly less fullness in women than in men. Hence, men required a larger meal load and stronger homeostatic sensations to achieve satisfaction. The same pattern was observed at the level of full satiation: men ate more and still experienced positive well-being, whereas in women, well-being scores dropped below pre-meal level. The effect of gender on the ingestion experience suggests that other constitutive factors of the eater may also influence responses to meals.

## 1. Introduction

Ingestion of a meal induces a biological response primarily related to the digestive process. If something goes wrong, for instance the stomach does not relax properly to accommodate the meal, patients may experience symptoms such as epigastric discomfort, a very frequent and relevant clinical problem [1,2]. To investigate the potential mechanisms of meal-related symptoms, an armamentarium of techniques for the evaluation of gut function and perception was developed over the years [2]. Recently, this methodology—to measure gastric motor activity and sensitivity—has been applied for the evaluation of the normal response to meal ingestion, in part motivated by the growing interest in the processes of food production and consumption [3].

Under normal conditions, meal ingestion is associated with a pleasant sensory experience, involving sensations related to homeostasis, e.g., satiety, fullness, with a hedonic dimension [3,4,5]. Responses to a meal, including the sensory experience, involve a series of events taking place, not only before and during ingestion, but also after meal completion during the post-prandial period.

These responses depend on the characteristics of the meal [6,7] and the responsiveness of the eater. Recent studies have shown that the responsiveness of the eater may be influenced by a series of conditioning mechanisms, i.e., inducible factors. For instance, a meal pre-load reduced appetite and modified the response to a subsequent meal by increasing satiation/fullness, but reducing satisfaction [8]. Conversely, a cognitive/sensory intervention increased post-prandial satisfaction, with lower intensity of homeostatic sensations [9].

In this context, we hypothesized that the responses to a meal also depend on inherent characteristics of the eater, i.e., constitutive factors. The aim of this proof-of-concept study was to determine whether and to what extent gender, as a constitutive factor, determines the response to a probe meal. More specifically, the tolerance of the meal, the sensory experience during the process of ingestion, and the post-prandial response. For that purpose, the probe meal was individualized by stepwise administration up to the level of full satiation, as the upper limit of consumption. In this study the specific contribution of sex(biology) versus gender (roles) in response to meal ingestion was not investigated, and the term gender is used in a broad sense to encompass the differences between women and men.

## 2. Material and Methods

### 2.1. Participants

Twenty healthy non-obese, non-dieting, and weight-stable subjects (10 women and 10 men), between 18 and 70 years of age, and without any history of gastrointestinal symptoms were recruited by public advertising to participate in the study. Exclusion criteria were chronic health conditions, use of medications (except occasional use of nonsteroidal anti-inflammatory drugs (NSAIDs) and antihistamines), history of anosmia and ageusia, current dieting or any pattern of selective eating such as vegetarianism, alcohol abuse, and the use of recreational drugs; due to its potential conditioning effect, prior obesity was also one of the exclusion criteria. Absence of current digestive symptoms was verified using a standard abdominal symptom questionnaire (no symptom >2 on a 0–10 scale). Psychological and eating disorders were excluded using the following tests: Hospital Anxiety and Depression scale (HAD), Dutch Eating Behavior Questionnaire (DEBQ—emotional eating, external eating, restrained eating), and the Physical Anhedonia Scale (PAS). Candidates were asked whether they liked the test meals (see below), and those who did not were excluded. In order to reduce variations in gut function related to the menstrual cycle, women were studied during the follicular phase of the menstrual cycle (days 5–15).

The research was conducted according to the Declaration of Helsinki. The study’s protocol had been previously approved by the Institutional Review Board of the University Hospital Vall d’Hebron, and all participants gave written informed consent.

### 2.2. Experimental Design

This study was a single-centre, parallel randomized study performed between September 2017 and January 2018. The study investigated the effect of gender on the responses to meal ingestion. The primary outcome was meal tolerance, measured as the amount of meal ingested up to the level of maximal satiation. The study’s protocol was registered with the ClinicalTrials.gov NCT03408977. All co-authors had access to study data, reviewed, and approved the final manuscript.

### 2.3. Randomization and Masking

To prevent time bias, men and women were studied in random order determined by a computer-generated randomization list. Participants were blind to the specific aim and primary outcome of the study.

### 2.4. General Procedure

**Figure 1:**
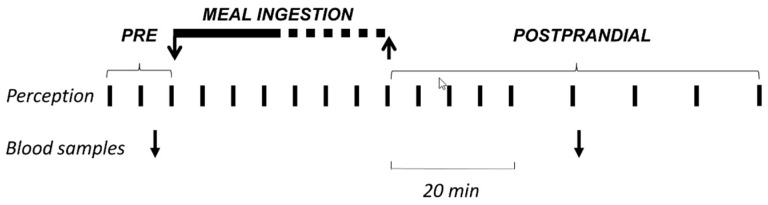
Experimental procedure. A comfort meal was administered at a fixed rate (150 Kcal/6 min) up to full satiation, while measuring the sensory expense at regular intervals.

Each participant was studied once. Participants were instructed to refrain from strenuous physical activity the day prior to the study, to refrain from smoking on the study day, to consume only a standard breakfast (tea or coffee, regular or decaffeinated depending on their habits, with semi-skimmed milk and biscuits; 181 Kcal, 5 g lipids, 27 g carbohydrate, 7 g protein) at home after overnight fasting, and to report to the laboratory where the probe meal was administered 4 h after breakfast. Studies were conducted on participants sitting alone with one investigator in a quiet, isolated room. Participants were instructed to eat up to the level of maximal satiation (see Probe meal). Perception was measured before, during, and after meal ingestion (see Perception measurements). Venous blood samples were taken immediately before, and 30 min after the Probe meal (Figure 1).

### 2.5. Probe Meal

In order to elicit a positive hedonic response, an assorted and attractive comfort meal was designed through a series of preliminary studies (Table 1). The probe meal was administered stepwise at a fixed rate: every 6 min an assorted meal serving was presented on a tray. Each portion consisted of fatty liver duck (Foie gras entero de pato mi-cuit, El Corte Inglés, Larrabezua, Spain; 10 g), toasts (Mini Tostas, Bimbo, Barcelona, Spain; 3 g), cheese (Queso emmental en lonchas, El Corte Inglés, Madrid, Spain; 10 g), potato chips (Patatas fritas clásicas 100% aceite de oliva, LAY’S, Vitoria, Spain; 5 g), salted peanuts (Cacahuetes fritos y salados, Borges, Reus, Spain; 2 g) and soft drink (Coca-Cola clásica, Coca-Cola, Madrid, Spain; 28 mL). At the end of each ingestion step, perception was measured. Ingestion was continued until maximal satiation (score 5 on a −5 to +5 score scale; see below). The eating rate was decided based on preliminary studies searching for a pleasant rate.

### 2.6. Perception Measurements

Five 10 cm scales graded from −5 to +5 were used to measure: (a) meal wanting (negative/eager), (b) meal liking (very disagreeable/very agreeable), (c) hunger/satiety (extremely hungry/completely satiated), (d) digestive well-being (extremely unpleasant sensation/extremely pleasant sensation), and (e) mood (negative/positive); two additional 10 cm scales graded from 0 (not at all) to 10 (very much) were used to measure: (f) abdominal bloating-fullness, and (g) discomfort-pain. Subjects received standard instructions on how to fill-out the scales [10]. The wanting scale was only scored at the presentation stage of each meal serving. The liking scale was only scored at the end of each ingestion step. The rest of the scales were scored: (a) at 5 min intervals 10 min before the meal, (b) at the end of each ingestion step, (c) at 5 min intervals during the first 20 min after ingestion, and (d) at 10 min intervals up to 60 min after finishing the probe meal (Figure 1). It has been previously shown that these scales detect consistent and reproducible differences in post-prandial sensations induced by various conditioning factors [3,4,5,6,7,8,9], and that perception measurements correlate with changes in circulating metabolites [10,11] and with some objective parameters of brain function measured by functional magnetic resonance [12].

### 2.7. Analytical Procedures

Once extracted, blood samples were immediately placed in ice. After completing the study, the samples were centrifuged for 15 min at 4 °C and 1500 *g* to separate blood components. Using 1 mL pipettes, samples of plasma and serum were placed in Eppendorf tubes and stored at −40 °C.

Routine laboratory techniques were used to measure insulin levels (AU5800 spectrophotometry system, Beckman Coulter, Brea CA, USA). GLP-1 was measured by enzyme-linked immunosorbent assay (ELISA) (GLP-1 Total ELISA Kit; Millipore, Billerica MA, USA).

The metabolomic analysis was performed using nuclear magnetic resonance (NMR), as previously described [10,11]. In brief, the concentration of the three different classes of lipoproteins (VLDL, LDL, and HDL), and their composition (content of cholesterol, triglycerides and large, medium and small particles) were determined [13]. A target set of 14 low molecular weight metabolites (LMWMs) was identified and quantified in the 1D Carr-Purcell-Meiboom-Gill spectra using Dolphin [14]. Each metabolite was identified by checking for all its resonances along the spectra, and then quantified using line–shape fitting methods on one of its signals [15]. Validation of metabolite identification was assisted by statistical total correlation spectroscopy (STOCSY) [16].

### 2.8. Statistical Analysis

Statistical analysis was performed using the Stata Software for Windows, (StataCorp. 2017. Stata Statistical Software: Release 15. College Station, TX: StataCorp LLC) and MetaboAnalyst 4.0 [17].

Considering a 20% difference in meal tolerance (Kcal to maximal satiation) biologically relevant, a sample of 20 subjects (10 women and 10 men) was estimated necessary to discriminate between the two groups with a power of 80% and a significance level of 5%.

In each group, mean and standard error (SE) of the measured variables were calculated. The Kolmogorov–Smirnov test was used to check the normality of data distribution. Parametric normally distributed data were compared by Student’s *t*-test for paired or unpaired data; otherwise, the Wilcoxon signed rank test was used for paired data, and the Mann–Whitney *U* test was used for unpaired data. The association of parameters was analyzed using Pearson’s test.

Temporal responses to meal ingestion were analyzed using one-way ANOVA for repeated measurements (10 min pre- and 60 min post-prandial period); when the ANOVA was significant, post-hoc comparisons between time-points were performed by applying the Sidak multiple comparison correction procedure. Comparisons between groups (men vs. women) were performed using repeated measures ANCOVA (dependent variable: post-prandial sensations scores; between and within subject’s factors: gender and time, respectively; covariate: pre-meal scores) [18].

Multivariate discriminant analysis of metabolomic data (standardized concentrations) was performed using an unsupervised classification by Principal Component Analysis (PCA), and supervised orthogonal partial least squares discriminant analysis (OPLS-DA).

Differences were considered significant at a *p* value <0.05.

## 3. Results

### 3.1. Demographics

Participants were 22–37 years of age with 19.7–24.7 Kg/m^2^ body mass index range without differences between women and men. Body weight was 61 ± 2 Kg in women and 75 ± 3 Kg in men (*p* = 0.003). All participants had normal bowel habits and scored Hospital Anxiety and Depression scale (HAD), Physical Anhedonia Scale (PAS), and Dutch Eating Behavior Questionnaire (DEBQ) within the normal range in all domains. All participants completed the studies and were included for analysis.

### 3.2. Baseline Conditions

**Figure 2:**
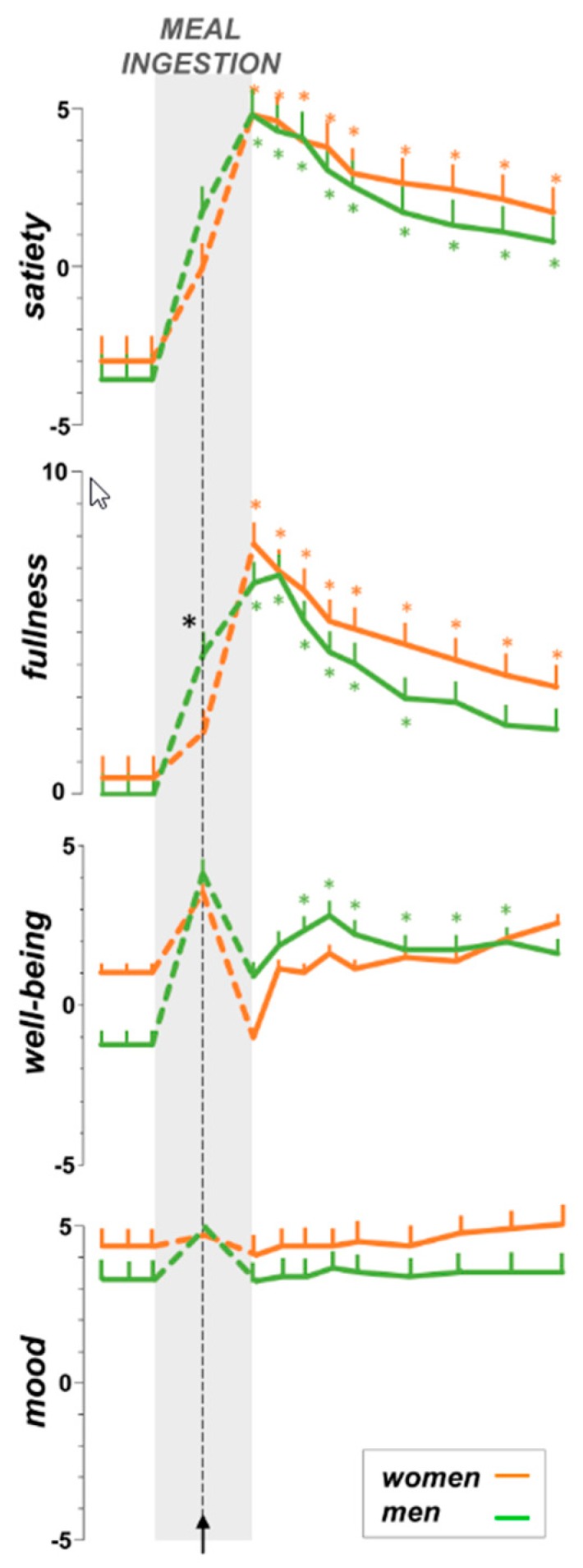
Effect of gender on meal-related sensations. Sensations were scored on 10 cm scales. During meal ingestion, well-being increased up to a peak and then progressively decreased until full satiation. No statistical differences in post-prandial sensations between groups were detected by Analysis of Covariance (ANCOVA), but the post-prandial well-being measured as the area under the curve, was significantly larger in men (*p* = 0.047). Temporal responses to meal ingestion analyzed using one-way Analysis of Variance (ANOVA) for repeated measures; asterisks indicate significant differences from pre-meal values by post-hoc comparisons (*p* < 0.05 applying the Sidak correction procedure for multiple comparisons).

Before the Probe meal (baseline fasting period), subjects reported hunger and positive mood in both groups alike, without fullness/bloating or discomfort/pain (Figure 2). Baseline well-being scores were higher in women than men (*p* = 0.046).

### 3.3. Sensations during Meal Ingestion

**Figure 3:**
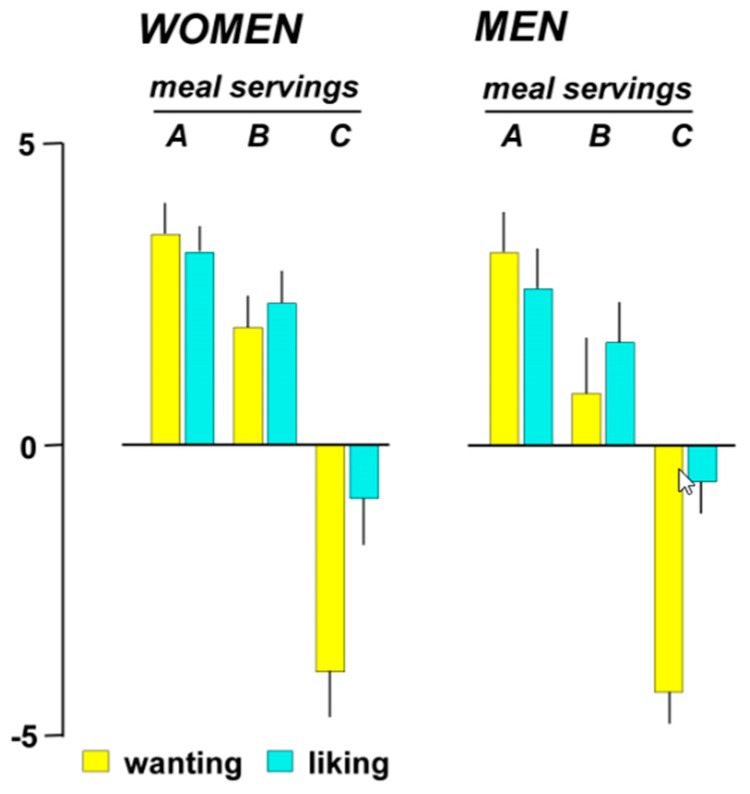
Meal-wanting and liking during meal ingestion. Data correspond to: (**A**) the first meal serving, (**B**) the serving that induced maximal well-being, and (**C**) the serving that induced full satiation (last serving).

At initial presentation, the meal was attractive with positive wanting scores, similar for both women and men (Figure 3). Wanting scores progressively declined down to negative values at the last ingestion step, i.e., wanting score at presentation of the serving that induced maximal satiation was rated −3.8 ± 1.0 score in women and −4.1 ± 0.6 score in men; *p* ≤ 0.004 vs. first step for both; *p* = 0.793 between groups).

All participants liked the first plate of the meal. Liking of the first plate correlated with wanting scores before eating it (R = 0.73; *p* < 0.001). Afterwards liking gradually declined down to neutral by the end of the meal (*p* ≤ 0.100 vs. first step for both groups; *p* = 0.750 between groups; Figure 3).

During the ingestion period, baseline hunger declined and the hunger/satiation axis progressively shifted towards maximal satiation by the end of the meal; the upper limit of the dose-response test, both in women and men (Figure 2). The amount of food tolerated was significantly smaller in women than in men (945 ± 59 Kcal vs. 1575 ± 147 Kcal, respectively; *p* < 0.001). No significant correlations were found between food tolerance and body mass index (BMI) (R = 0.21; *p* = 0.176) or weight (R = 0.41; *p* = 0.079). During meal ingestion the fullness sensation gradually increased, and by the end of the meal, fullness scores were similar in women and men (Figure 2).

Initially, meal ingestion significantly increased the sensation of digestive well-being. The amount of food that induced the well-being peak tended to be lower in women than in men, but the difference did not reach statistical significance (345 ± 71 Kcal vs. 660 ± 129 Kcal, respectively; *p* = 0.069); well-being scores approached the top of the scales in both groups, regardless of the differences in basal scores (Figure 2). At the point of maximal well-being, mood scores were also high, and an increase was observed in men (*p* = 0.046 vs. pre-meal); at that point, men experienced more fullness than women (*p* = 0.029) and satiation scores also tended to be higher (*p* = 0.123). After having reached the well-being peak, well-being decreased to a minimal level by the end of meal ingestion (Figure 2); well-being scores were below pre-meal level in women and above in men, and the changes from basal levels were significantly different between groups (*p* = 0.025).

**Figure 4:**
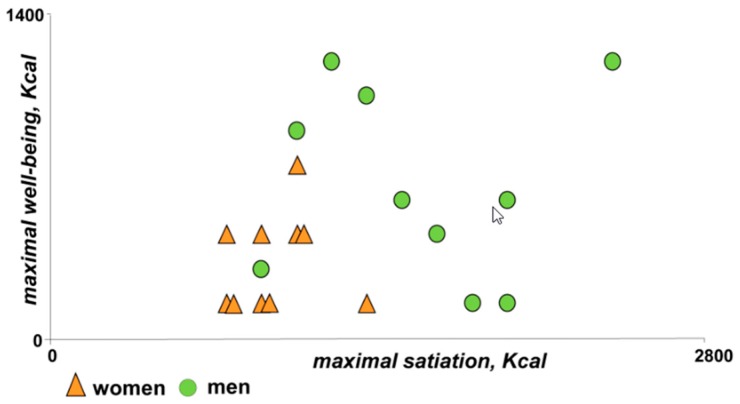
Relation between meal loads that induced maximal well-being and full satiation. Note discrimination between women and men.

The amount of food that induced the well-being peak did not correlate with the amount of food inducing maximal satiation (R = 0.22; *p* = 0.177); considering together the amount of food inducing maximal well-being and that inducing maximal satiation a good discrimination between women and by men was found (*p* = 0.002 by canonical lineal discriminant analysis; Figure 4).

All participants ingested at least 750 Kcal (5 meal servings); at this common maximal level, satiation was higher in women than in men (3.6 ± 0.4 score vs. 1.2 ± 0.7 score, respectively; *p* = 0.014); at this point, fullness tended to be higher in women (4.1 ± 0.7 score vs. 2.7 ± 0.9 score, respectively; *p* = 0.235), whereas well-being (1.3 ± 0.7 score vs. 2.1 ± 0.5 score, respectively; *p* = 0.344) and mood (3.1 ± 0.5 score vs. 3.4 ± 0.3 score, respectively; *p* = 0.606) tended to be lower than in men, but the differences were not statistically significant.

### 3.4. The Post-Prandial Experience

#### 3.4.1. Homeostatic Sensations

During the post-prandial period both women and men reported satiety and fullness (main time-effect *p* < 0.001 for all; Figure 2). These sensations gradually decayed; however, post-hoc comparisons showed that the scores remained significantly higher than pre-meal scores through the 60 min post-prandial period, except for fullness in men during the last 30 min of the observation period. No significant differences between women and men were observed for post-prandial satiety [main gender-effect F (1, 17) = 1.97; *p* = 0.179] and fullness [F (1, 17) = 0.52; *p* = 0.486].

#### 3.4.2. Hedonic Responses

In men, the immediate drop in well-being at termination of the meal recovered and was followed by a post-prandial increase in the sensation of digestive well-being (main time-effect; *p* < 0.011). Post-hoc comparisons showed that by 15 min and up to the end of the post-prandial observation period, well-being scores were significantly higher than pre-meal scores (Figure 2). In women, the initial drop in well-being rapidly recovered, but not beyond pre-meal levels (main time-effect; *p* = 0.061); no significant gender effects were detected by ANCOVA [F (1, 17) = 0.38; *p* = 0.548], but the area under the curve during the post-prandial period (over pre-meal levels) was significantly smaller in women than in men (*p* = 0.047). Overall, post-prandial well-being correlated with initial wanting (R = 0.64; *p* = 0.001) and first liking (R = 0.53; *p* = 0.008). The Probe meal did not induce significant effects on post-prandial mood in either women or in men (main time-effect; *p* = 0.255 and *p* = 0.633, respectively) and no gender effects were observed [F (1, 17) = 1.01; *p* = 0.329].

### 3.5. Hormonal Response

**Figure 5:**
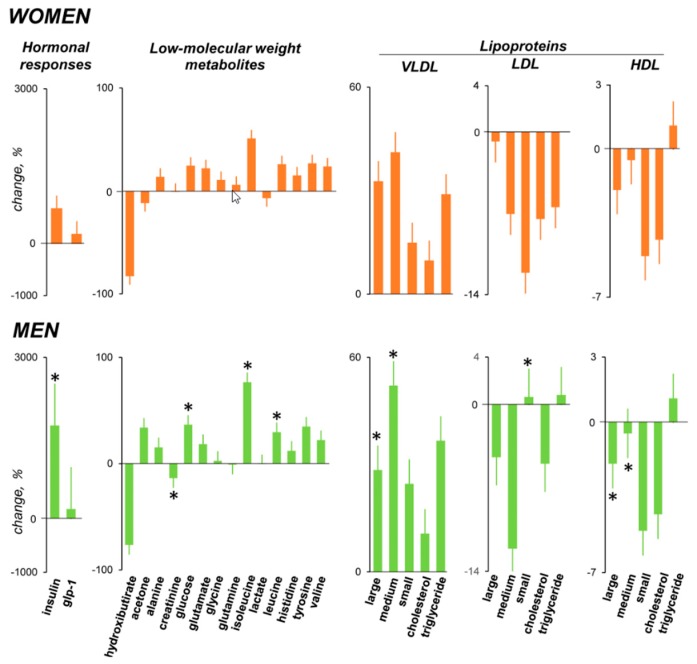
Metabolomic response to the meal. Data are changes from basal. Note, a more pronounced response in men than in women (* *p* < 0.05 men vs. women), possibly related to their larger meal load.

Meal ingestion induced a significant increase in insulin, and the responses were significantly smaller in women than in men (*p* = 0.003; Figure 5). No significant changes in GLP1 after meal ingestion were detected.

### 3.6. Metabolomic Response

#### 3.6.1. Low-Molecular Weight Metabolites

Meal ingestion was associated with a change in the spectrum of plasmatic low-molecular weight metabolites. Sample classification using an unsupervised PCA and OPLS-DA model correctly discriminated between the pre- and post-prandial state in both groups (data not shown). The main metabolites responsible for the post-prandial switch also exhibited significant post- versus pre-prandial differences by univariate analysis. To overcome the relatively small group sizes, the data from women and men were pooled together, and analyzing the responses in the whole set of participants, the levels of alanine, glucose, isoleucine, leucine, tyrosine, and valine increased after the meal, while 3-hydroxibutirate decreased (*p* ≤ 0.038 for all; pooled data not shown). Post-prandial changes tended to be smaller in women than in men (Figure 5).

#### 3.6.2. Lipoprotein Profile

The probe meal induced a consistent change in the lipid profile (Figure 5). Considering altogether the responses in the whole set of participants (i.e., pooling the data in women and men), large, medium, small and triglyceride VLDL significantly increased; by contrast, medium LDL significantly decreased (pooled data not shown). These changes tended to be smaller in women than in men (Figure 5).

## 4. Discussions

Our data indicate that gender influences the response to a comfort meal, and particularly, the relation between homeostatic and hedonic sensations.

Homeostatic and hedonic sensations are dissociable, e.g., depending on various conditioning factors, post-prandial satiety and fullness may have a pleasurable or aversive dimension [3], and our study shows that gender also plays a role. Initially, ingestion of the comfort meal induced a positive hedonic response, and satisfaction increased up to a peak of digestive well-being. However, the relation of homeostatic and hedonic sensations is bimodal, and from the point of maximal satisfaction, the relation became inverse: overconsumption of the meal kept increasing satiation, whereas digestive well-being gradually decreased down to a nadir at the point of full satiation.

The amount of food consumed at the well-being peak tended to be lower in women than in men, and interestingly, at equal levels of satisfaction, the fullness sensation was significantly lower in women. Hence, in men a larger meal load and stronger homeostatic sensation were required to achieve full satisfaction. The same pattern was observed at the level of full satiation: men ate more and still experienced positive well-being, whereas in women well-being dropped to negative scores below pre-meal levels. Well-being scores before the meal were slightly higher in women than in men, the difference reached statistical significance but has not been replicated in subsequent studies.

The results of the present study may explain gender differences in eating behaviors. Indeed, on a standard diet adjusted to meet individual energy requirements, women reported less appetite and more post-prandial satiety (less hunger) than men [19,20]. Where one of the main factors to stop eating in women is the extinction of the pleasure response (the food stops tasting good) [21].

In normal conditions, eating behavior matches energy requirements, which are different in women and men. Women have lower resting metabolic rates [22] and consume less energy per kilogram lean mass [23,24]. The reasons for these gender differences are not known, but may relate to sex steroids, insulin resistance, and other hormones, such as leptin [23,25]. Lower body weight in women might also play a role, but it has been shown that gender-effects persist after controlling for fat-free mass [22].

Our data are concordant with previous observations using challenge drink tests [26,27,28]. These studies measured the volume of meal tolerated up to the level of discomfort, and under these conditions, women experienced discomfort with lower loads than men. Gender differences were detected using water or nutrient drinks at high ingestion rates, but not at low rates [28]. Conceivably, lower tolerance of the challenge drink tests in women reflects higher stomach sensitivity, because studies applying graded distension of the stomach by means of an air-filled bag, showed that at the same distending levels, perception is higher in women, but the pressure/volume relation, i.e., gastric compliance, is equal to that in men [29]. Gastric distension in these studies was standardized by means of an electronic pump that maintains fixed pressure levels within an intragastric bag, i.e., gastric barostat [30].

During fasting, the stomach is contracted, and ingestion of a meal induces a reflex relaxation to accommodate the ingested volume load (gastric accommodation reflex). Subsequently, the stomach progressively re-contracts during the post-prandial period, and this re-contraction gently forces gastric chime into the duodenum [31]. This process is regulated by a complex series of reflexes [1,2]. Some data indicate that post-prandial gastric re-contraction and gastric emptying take longer in women than in men [29,32,33]. These effects may explain the trend towards more persistent post-prandial sensation in women as compared to men in our study.

As in previous studies, meal ingestion affected the profile of circulating metabolites [10,11]. The increase in GLP-1 was of similar magnitude as previously observed [10], but the increase was not statistically significant, probably due to the large inter-individual variability. The metabolomic response was more pronounced in men, and this was probably related to their larger meal load. Hence, while metabolomic responses are related to meal load, post-prandial sensations depend on individual factors, and gender plays a determinant role.

Our study shows gender differences in response to a comfort meal, and these data are relevant for understanding differences in eating behaviors. However, from a broader perspective, these data support our hypothesis on the role of the eater’s inherent characteristics, i.e., constitutive factors, on the post-prandial experience. This proof-of-concept study suggests that the identification of other constitutive factors may help to individualize dietary interventions for specific goals. This opens a path in the growingly popular field of gastronomy; it is tantalizing to speculate that the specialized chef after studying the guest, may tailor the menu for a wonderful eating experience. These data may also have more pragmatic applications for health care purposes. Individualized diets may help patients with different conditions ranging from eating and metabolic disorders, to patients with specific nutritional requirements, and those with meal-related symptoms.

## Figures and Tables

**Table 1:** Probe meal: content per serving *.

	Total	Total	FAT	PROT	CHO
	g	kcal	g	g	g
fatty liver duck ^+^	10	53.1	5.5	0.8	0.1
toast	3	12.2	0.1	0.4	2.3
cheese	10	34.7	2.7	2.6	0.0
potato chips	5	25.6	1.6	0.3	2.5
peanuts	2	12.8	1.1	0.6	0.2
cola drink	28	11.8	0.0	0.0	3.0
per serving	58	150.0	11.0	4.6	8.0

PROT: proteins, CHO: carbohydrates; ^+^ Foie gras mi-cuit; * one serving (55 mL) administered every 6 min up to full satiation.
